# Microbial Bioherbicides Based on Cell-Free Phytotoxic Metabolites: Analysis and Perspectives on Their Application in Weed Control as an Innovative Sustainable Solution

**DOI:** 10.3390/plants13141996

**Published:** 2024-07-22

**Authors:** Diego Ocán-Torres, Walter José Martínez-Burgos, Maria Clara Manzoki, Vanete Thomaz Soccol, Carlos José Dalmas Neto, Carlos Ricardo Soccol

**Affiliations:** Department of Bioprocess Engineering and Biotechnology, Federal University of Paraná, Curitiba 81531-990, Brazil; diego.ocan@ufpr.br (D.O.-T.); mariamanzoki@ufpr.br (M.C.M.); vanetesoccol@gmail.com (V.T.S.); cardalmas@gmail.com (C.J.D.N.)

**Keywords:** bioherbicide, bioprocesses, phytotoxin, metabolite, weed, patents

## Abstract

Weeds cause significant agricultural losses worldwide, and herbicides have traditionally been the main solution to this problem. However, the extensive use of herbicides has led to multiple cases of weed resistance, which could generate an increase in the application concentration and consequently a higher persistence in the environment, hindering natural degradation processes. Consequently, more environmentally friendly alternatives, such as microbial bioherbicides, have been sought. Although these bioherbicides are promising, their efficacy remains a challenge, as evidenced by their limited commercial and industrial production. This article reviews the current status of microbial-based bioherbicides and highlights the potential of cell-free metabolites to improve their efficacy and commercial attractiveness. Stirred tank bioreactors are identified as the most widely used for production-scale submerged fermentation. In addition, the use of alternative carbon and nitrogen sources, such as industrial waste, supports the circular economy. Furthermore, this article discusses the optimization of downstream processes using bioprospecting and in silico technologies to identify target metabolites, which leads to more precise and efficient production strategies. Bacterial bioherbicides, particularly those derived from *Pseudomonas* and *Xanthomonas*, and fungal bioherbicides from genera such as *Alternaria*, *Colletotrichum*, *Trichoderma* and *Phoma*, show significant potential. Nevertheless, limitations such as their restricted range of action, their persistence in the environment, and regulatory issues restrict their commercial availability. The utilization of cell-free microbial metabolites is proposed as a promising solution due to their simpler handling and application. In addition, modern technologies, including encapsulation and integrated management with chemical herbicides, are investigated to enhance the efficacy and sustainability of bioherbicides.

## 1. Introduction

Weeds are plants that adversely affect crops due to various characteristics that enable them to grow faster and more efficiently, even in adverse environments. They can deplete soil resources, thereby limiting crop growth, which subsequently poses a problem for the agricultural sector. This situation leads to increased production costs and decreased profits in the market [1]. To provide an example of the magnitude of the weed problem globally, it has been estimated that in India and the USA, weeds have caused losses of USD 11 billion and USD 17.2 billion per year, respectively, specifically in some significant crops such as soybeans and dry beans [2,3,4].

To control the incidence of this type of unwanted plants, chemical products called herbicides have been applied. Herbicides interact with different sites of action of plants, affecting or inhibiting the production of certain essential components necessary for their metabolism [5]. Glyphosate, for example, is a widely distributed herbicide that inhibits the enzyme EPSPS (5-enolpyruvylshikimate-3-phosphate synthase), which is indispensable in the shikimic acid pathway when it comes to producing essential amino acids [6]. Until 2019, approximately 2 million tons of pesticides have been used in the world, of which 47.5% were herbicides. China, the United States, Argentina, Thailand, Brazil, Italy, France, Canada, Japan, and India are the countries with the highest demand for these products [7]; likewise, glyphosate is known to be the most commercial herbicide worldwide [8]. In view of this, the constant and uncontrolled use of herbicides has led to the development of resistance mechanisms by weeds, especially to chlorsulfuron and glyphosate [9]. In addition, herbicides have had a negative impact on the environment because they are highly complex to degrade and persist in aquatic bodies and other substrates, becoming recalcitrant products that could potentially harm human health [10,11].

Moreover, in response to the challenge posed by the development of herbicide resistance, synthetic herbicides with novel modes of action have been investigated and developed. One such example is cinmethylin, which inhibits acyl-ACP thioesterase (FAT), a crucial enzyme in the lipid biosynthesis pathway in plants [12]. Another example is cyclopyrimorate, which targets homogentisate solanesyltransferase (HST), an enzyme essential for the biosynthesis of plastoquinone, a vital cofactor in photosynthesis [13]. Additionally, the inhibition of solanesyltransferase (HST), another enzyme involved in the biosynthesis of plastoquinone, and the targeting of tetflupyrolimet to dihydroorotate dehydrogenase (DHODH), an enzyme situated on the outer surface of the inner mitochondrial membrane and involved in pyrimidine biosynthesis, have been explored [14]. However, the high homology between crops and weeds, particularly at herbicide action sites, complicates the discovery process. Furthermore, the lack of specificity may adversely affect crops [15,16].

To counteract this situation, several alternatives have been developed, such as the use of living organisms with the ability to produce specific phytotoxic compounds, called bioherbicides or biological herbicides [17]. These weed-control agents include plants, bacteria, fungi and viruses [18]. Among them, bacteria and fungi have been the most extensively utilized, not only in research but also at the industrial level. This is largely due to their greater ease of production on an industrial scale, as well as other factors, such as the specificity, sustainability, and control efficiency [19]. This has resulted in the production of commercial products with high efficiency and, most importantly, eco-sustainability [20].

One of the primary advantages of using microbial bioherbicides for agricultural weeds is their significantly lower discovery and development costs compared to synthetic herbicides. The costs of synthetic pesticides have increased over the past decades due to factors such as the diminishing returns from extensive compound screening, a more competitive market, and heightened safety requirements. The development of synthetic herbicides involves extensive chemical synthesis and large-scale efficacy and safety testing. In contrast, developing a bioherbicide from an endemic plant pathogen does not require extensive chemical synthesis or the same level of environmental and toxicological testing. Consequently, most microbial bioherbicides have been developed by small companies rather than major synthetic pesticide firms. Additionally, the regulatory approval costs for microbial biopesticides in the USA are considerably lower than those for synthetic pesticides. Although the approval costs in the European Union are higher than in the USA, they are still lower than those for synthetic pesticides [21].

However, bioherbicides have some shortcomings that hinder their commercial development, as evidenced by the limited number of products currently available on the market [21,22,23]. Despite this, extensive research has been conducted in recent years to address these issues. The application of cell-free phytotoxic metabolites has garnered significant attention because it increases the efficiency of weed inhibition and limits the persistence of the product in the environment, thereby reducing its spread to non-target crops [24,25,26,27,28]. Other important aspects of cell-free phytotoxic metabolites include the formulation and application of these products, as well as the use of industrial residual substrates as fermentation process substrates, which reduces production costs [29].

In this context, this article presents a complete review of microbial bioherbicides, with a primary focus on their application as cell-free phytotoxic metabolites. It also includes an analysis of the status and historical patents of biological herbicides based on bacteria and fungi, produced both commercially and for research purposes. Lastly, this article reviews critical aspects that limit their development as commercial products. The novelty of the present literature review lies in analyzing the context of bioherbicides, both scientifically and industrially, in order to identify the possible obstacles that hinder their development as products that actually reach the market significantly.

## 2. Development and Production of Microbial Bioherbicides Based on Cell-Free Metabolites

The development of a typical microbial herbicide, that is, microbial bioherbicides based on microbial cultivation, may include two main stages: screening and isolation of the microorganism with specific phytotoxic capability, and large-scale production for subsequent formulation and application. However, for the development of microbial bioherbicides based on cell-free metabolites, in addition to the aforementioned processes, additional steps will be required for the extraction and purification of the metabolites of interest, involving considering the chemical nature of each of them, which could either facilitate or complicate the process.

**Figure 1:**
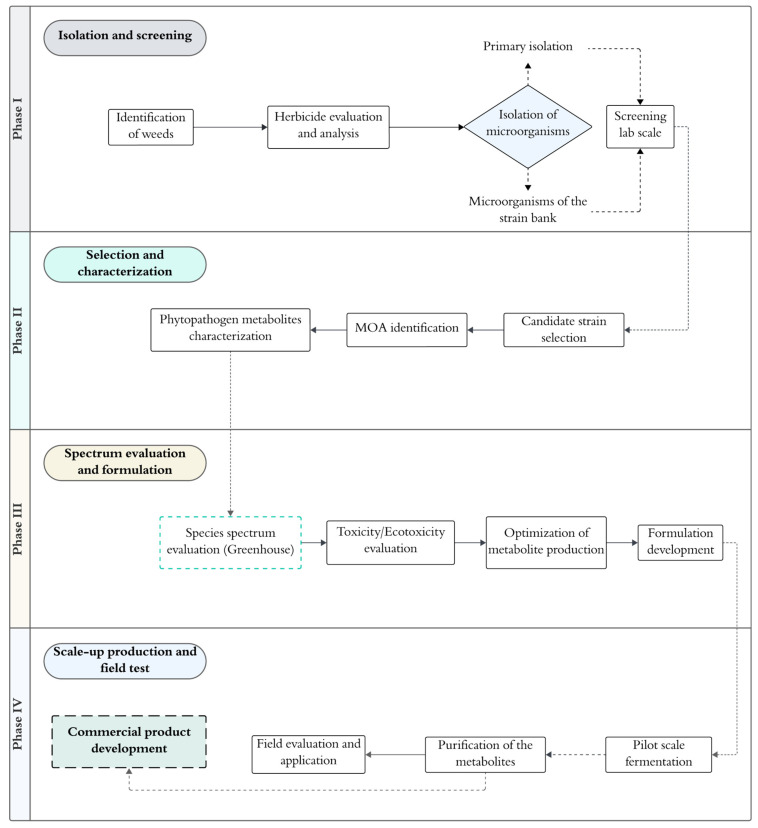
Flow chart for the development of a microbial bioherbicide based on metabolites produced by bacteria or fungi. Four main development phases take place for the successful establishment of a new bioherbicide.

The phases involved in the development of a bioherbicide based on metabolites produced by microorganisms such as bacteria and fungi can be described in four phases (Figure 1). The first phase consists of the isolation and screening of microorganisms capable of acting against a specific weed species. This process begins with the identification of weed species present in a given environment and the evaluation of chemical herbicides associated with their control to understand the possible mechanisms of action or resistance to herbicides [9]. Subsequently, primary sampling and isolation must be conducted to select candidate microorganisms as bioherbicides. Samples can be collected from the damaged areas of a weed infected by a phytopathogen or from the rhizosphere level of soils where crops are affected by weeds [30,31]. If previously isolated strains from a reference strain bank are available, they can also be used for subsequent screening, which will be carried out in Petri dishes using seeds of the target weed and evaluating various responses, such as germination, shoot size, root size, etc. [32].

In the second phase of bioherbicide development, the strain or strains with the best results after screening will be selected and subjected to a metabolite characterization process. In this way, the mode of action (MOA) of the bioherbicide can be recognized. The equipment necessary to perform a metabolite profile might include sophisticated equipment such as mass spectrometry or the use of computational techniques like molecular docking [33]. In the third phase of the process, the goal will be to evaluate the spectrum of weed species targeted by the bioherbicide product and its efficacy [34]. Up to this phase, the evaluation can be carried out using both isolated metabolites and microbial culture. Furthermore, the evaluation should be conducted in greenhouses to collect data more closely related to a natural habitat. Toxicity or ecotoxicity assays will also be necessary, primarily using crops related to the target weed to determine the selectivity level of the bioherbicide product [35]. After these tests, the production process of the herbicide will be optimized to improve the metabolite yield, considering multiple factors and the most recent literature. Subsequently, the product formulation will be developed, which will undergo more comprehensive evaluation to assess its efficacy against a spectrum of weed species and its toxicity to crops [36,37,38]. Finally, in the fourth phase of development, the bioherbicide will be produced on a large scale through fermentation, and the metabolites will subsequently be purified for field testing to corroborate their efficacy. Only then can the product be registered, patented, and finally commercialized.

At the production level, one of the most relevant stages of this process is fermentation, which depends on multiple variables to obtain an optimized product in terms of the yield. This is crucial because, currently, one of the main disadvantages of biological herbicides is the high production cost compared to chemical herbicides [29]. In a techno-economic analysis presented by Mupondwa [39], it was estimated that the total capital investment for a fermentation plant with two 33,000 L fermenters and a production of 3602 tons per year would be USD 17.55 million, with an annual operating cost of USD 14.76 million. Moreover, the payback period is less than one year, with a net present value (NPV) of 7%, which is considered profitable. However, this could be improved by employing different strategies at the production level.

A viable strategy involves the use of alternative substrates, particularly agro-industrial residues. Research into these residues as potential sources of carbon or nitrogen could represent an economically feasible approach to optimizing bioherbicide production, as corroborated by previous studies [40,41,42]. Additionally, several strategies can be implemented, including process optimization through statistical methodologies, the use of microbial consortia to broaden the range of target weeds, the selection of appropriate fermenter types and operational modes, among other potential avenues [11,43,44].

For this reason, the use of residues of different origins as sources of carbon and nitrogen can be explored as an economically viable strategy for optimizing bioherbicide production. Other strategies can be applied, such as process optimization using statistical tools, the use of microbial consortia to increase the range of target weeds, the correct choice of fermenter type and mode of operation, among others.

Below, we will describe the upstream and downstream stages in the production process for a microbial herbicide based on cell-free metabolites.

### 2.1. Upstream Process

To facilitate the development of upstream processes for microbial bioherbicides based on cell-free metabolites production, it is advisable to utilize a bank of pre-selected and well-characterized microbial strains. It is imperative that these strains demonstrate high efficacy, as validated through greenhouse or field trials, in controlling one or more target weeds, depending on the intended capabilities of the microorganism. Additionally, optimizing the fermentation conditions—such as nutritional, chemical, physical, and biological factors—is crucial to accelerate the fermentation process. Prior to scaling up to fermentation bioreactors, microbial inoculum must exhibit stringent quality in terms of viability [45]. Furthermore, the quantity of inoculum should be predetermined using mathematical models and microbial growth kinetics with established factors, aiming to minimize the initial latency phase and expedite exponential growth.

According to Bordin et al. (2021) [20], 60% of existing bioherbicides are derived from fungal (38%) and bacterial (16%) strains. For fungi, key fermentation considerations include optimizing the conditions for sporulation, light exposure as an abiotic stimulus, carbon source concentration, and incubation duration [46,47]. Conversely, bacteria, due to their faster metabolism, require shorter adaptation or fermentation periods and lower carbon source concentrations. However, increased agitation or airflow may be necessary, with submerged cultures being preferred. In contrast, solid-state cultures are favored for fungi [44,48].

Additionally, there is potential to develop inoculants comprising multiple microbial species—a microbial consortium. Studies indicate that microbial consortia often exhibit enhanced effectiveness in various biotechnological processes [49,50,51]. For bioherbicide production, this approach offers the advantage of diversifying phytotoxic metabolites, thereby broadening the spectrum of targeted weeds and enhancing the economic feasibility by consolidating production into a single fermentation batch rather than separate processes [52]. However, such approaches require rigorous laboratory testing to ensure the viability and minimize the antagonism among strains during fermentation, aiming to simulate synergistic interactions rather than inhibition [53].

Once the inoculants are prepared and the fermentation conditions optimized on a small scale, scaled-up microbial fermentation can commence for both bacteria and fungi. Notably, the choice of bioreactor type—tailored to the production objectives—is critical. Submerged fermentation (SmF) predominates in bioherbicide production, owing to its controllability and scalability [44]. For instance, Brun et al. (2016) [54] successfully produced a *Phoma* sp.-based bioherbicide for controlling *Cucumis sativus* var. *wisconsin* (cucumber) and *Sorghum bicolor* (sorghum) using stirred tank bioreactors, achieving up to 100% germination inhibition for both species. The optimal conditions included agitation rates of 40–60 rpm, aeration at 3 vvm, 10% (*v*/*v*) inoculum volume, and pH 6.0 for 7 days.

The commonly used bioreactors for SmF include stirred tank bioreactors and pneumatic bioreactors. Stirred tank bioreactors are favored industrially for their high volumetric mass transfer coefficients, facilitated by mechanical agitation via propellers or paddles, ensuring homogeneous gas dispersion and continual oxygen supply to microorganisms [55]. In contrast, pneumatic bioreactors, such as airlift bioreactors, utilize forced air or gas injection at the vessel base, providing controlled oxygen distribution with minimal shear [56]. Although less efficient for filamentous fungi, pneumatic bioreactors offer potential industrial optimization opportunities depending on the microbial requirements [57].

Solid-state fermentation (SSF) represents a viable alternative, simulating natural habitats with minimal free water in a solid matrix, often utilizing agro-industrial wastes as nutrient sources—an economically viable strategy for bioherbicide production [58,59]. For example, Bastos et al. (2017) [60] demonstrated improved bioherbicide activity against *Cucumis sativus* using *Diaphorte* sp. under SSF conditions, achieving significant reductions in the target height and dry weight compared to controls. Furthermore, Oliveira et al. (2019) [61] optimized cutinase production using SSF with *Fusarium verticillioides*, achieving maximum yields of 16.22 U/g. SSF can operate statically, with mechanical air circulation or air injection, with tray bioreactors being a practical choice due to their ease of assembly and control, facilitating gas diffusion and carbon dioxide removal [62]. However, challenges such as humidity control, scalability, and product purification complexities may limit its widespread adoption despite the potential yield advantages [48].

The selection of the fermentation mode—batch, fed-batch, or continuous—is pivotal and should align with the production objectives and metabolic efficiency requirements. While batch and fed-batch cultures are common for obtaining kinetic parameters, continuous fermentation offers advantages by maintaining constant broth input and output, minimizing the downtime in industrial production settings—an attractive proposition for producing phytotoxic metabolites in bioherbicides [29,63,64].

For addressing the economic constraints in bioherbicide production, utilizing industrial wastes as carbon or nitrogen sources presents a practical alternative. This approach has proven successful in various biotechnological applications, contributing to circular economy principles and waste valorization. For instance, Cavalcante et al. (2021) [42] utilized orange and shrimp peels in submerged fermentation of *Trichoderma koningiopsis* and *Rhizopus stoloniferse*, obtaining aqueous extracts with bioherbicidal potential against *Crocus sativus*. Similarly, Camargo et al. (2023) [40] utilized microalgae biomass from biogas digestate treatment to produce bioherbicides based on *Fusarium* and *Trichoderma*, achieving significant foliar damage (80–100%) in *Cucumis sativus*. Mitchell et al. (2003) [65] evaluated various carbon sources for optimizing sporulation medium for *Gloeocercospora sorghi*-based bioherbicide, highlighting bean brine as the optimal substrate for an effective response. In another study, Brun et al. (2016) [54] utilized corn mash liquor as a carbon and nitrogen source for *Phoma* sp.-based bioherbicide, demonstrating effective control of cucumber and sorghum.

### 2.2. Downstream Process

Once microbial cultivation has been completed, the subsequent step involves the recovery and concentration of the product to eliminate impurities and purify the biomolecule of interest. As previously mentioned, most bioherbicides are developed from microbial metabolites. Therefore, the initial focus should be on identifying the types of metabolites produced by the microbial species. For instance, phytotoxins such as cyclo-(proline-phenylalanine) or organic acids produced by *Xanthomonas retroflexus* are secondary metabolites found in the microbial supernatant, which are capable of inhibiting *Amaranthus retroflexus* L. (redroot pigweed). *Streptomyces* sp. produces anisomycin with high herbicidal activity against *Digitaria sanguinalis* and *Echinochloa crusgalli* (barnyard grass). Additionally, 4-hydroxy-3-methoxycinnamic acid, a secondary metabolite of the fungus *Pythium aphanidermatum*, inhibits the growth of *D. sanguinalis* [66,67,68]. These examples illustrate the diverse microbial metabolites acting as bioherbicides.

In the downstream stage, the initial step is the separation of the microbial biomass from the bioactive compounds, followed by metabolite concentration and purification. For solid-state fermentation (SSF), all the components attached to the solid substrate, including the biomass and metabolites, must be recovered. Bastos et al. (2021) [60] employed distilled water in a 1:10 ratio (*w*/*v*) with agitation at 100 rpm and 28 °C for 1 h to separate *Diaporthe* sp. biomass and its phytotoxic components from the solid substrate. The resulting broth was filtered and stored for later concentration.

For submerged fermentation (SmF), since both the biomass and metabolites are in a liquid medium, extraction is unnecessary. Given that most reported phytotoxic metabolites are extracellular, cell lysis for intracellular content release is not required [69]. Thus, the first downstream step in SmF is separating the cell biomass from the metabolites in the supernatant, typically using centrifugation or filtration with 0.45 μm membranes. Centrifugation, although more expensive, is recommended for smaller-scale production, whereas filtration is more viable for industrial-scale processes due to economic considerations [70].

At this stage, the product may exhibit high efficacy, but further purification may be needed to remove residues that could impact the bioherbicide performance. Metabolite concentration and purification can be achieved through filtration techniques or solvent extraction, as selected based on the metabolite’s physical and chemical properties. For example, Chaves Neto et al. (2021) [25] used flat polymeric ultrafiltration, microfiltration, and nanofiltration membranes to concentrate *Phoma dimorpha*-fermented broth, obtaining fractions with high phytotoxic potential against *Echinochloa* sp., *Amaranthus cruentus*, *Senna obtusifolia*, and *Bidens pilosa*. Similarly, Brun et al. (2016) [54] utilized various polarity solvents (methanol, ethanol, ethyl acetate) for liquid–liquid extraction of phytotoxic metabolites from *Phoma* sp. cultured in a stirred tank bioreactor, identifying pyrrolo[1,2-a]pyrazine-1,4-dione, hexahydro-3-(2-methylpropyl) as the compound with the highest herbicidal action against *Cucumis sativus* and *Sorghum bicolor*.

On an industrial scale, metabolite identification and purification are not yet fully developed, as commercial bioherbicides are typically formulated with live microbial cells. Thus, purification processes are primarily conducted at the laboratory scale. Chromatographic techniques are extensively used to analyze the phytotoxic metabolite composition. For example, the phytotoxin cyclo-(proline-phenylalanine) produced by *Xanthomonas retroflexus*, which inhibits the growth of *Amaranthus retroflexus*, was isolated by Li et al. (2007) using high-performance liquid chromatography (HPLC) and identified by gas chromatography–mass spectrometry (GC-MS) [66]. Similarly, Zang et al. (2013) purified 4-hydroxy-3-methoxycinnamic acid and two indole derivatives from the cell-free supernatant of *Pythium aphanidermatum* using HPLC coupled with a UV spectrophotometric detector and a reversed-phase Agilent C18 column, achieving 100% inhibition of *D. sanguinalis* root and coleoptile growth [67].

Advancing metabolite purification necessitates comprehensive preliminary studies or bioprospecting of the target metabolites, facilitated by modern in silico technologies such as molecular docking. These technologies enable the identification of specific phytotoxic metabolites and their potential target proteins in weeds, allowing simulation of interactions to determine the affinity and interaction energy. This approach aids in selecting the appropriate purification technique more accurately, reducing the additional costs and high production expenses in bioherbicide development [33].

Post-concentration and purification, the bioherbicide must be formulated appropriately based on the application method. For instance, solid formulations are more effective than liquid formulations when applied by spraying [71]. Formulations combined with adjuvants, such as surfactants, can enhance the metabolite permeability through plant cell walls [72]. Biogranular solid formulations using rice, wheat, soybean, and seed flours, as well as microemulsions, are promising alternatives for bioherbicide formulations based on fungal spores [35]. Finally, once the formulation is established, the product should be stored under appropriate, pre-established conditions, ready for research or commercial use.

**Figure 2:**
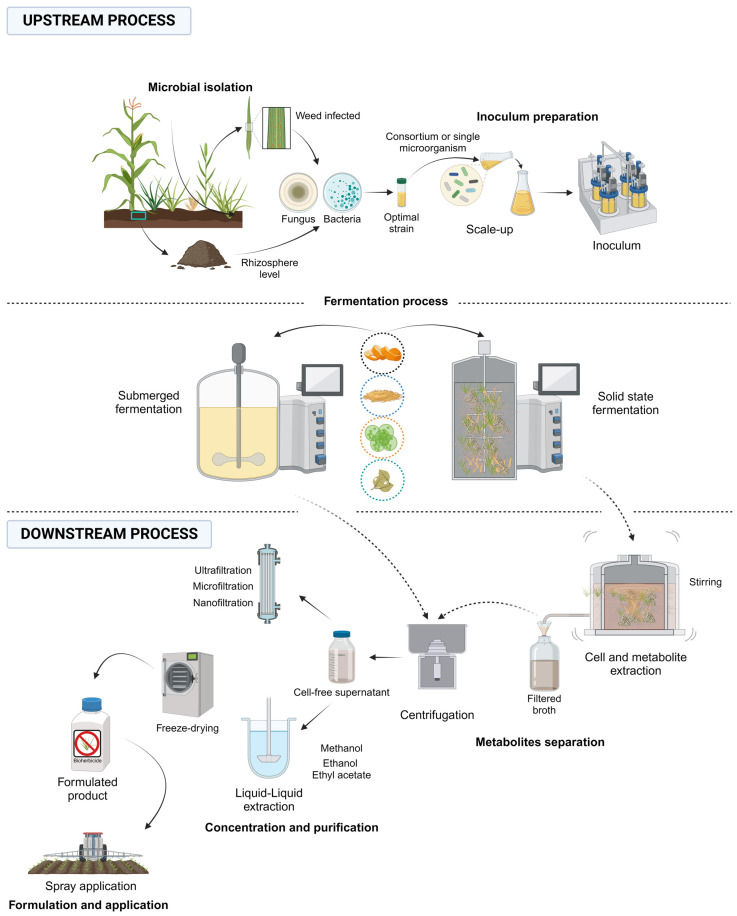
General scheme of the production of microbial bioherbicides based on cell-free metabolites of fungal or bacterial origin, mainly divided into the upstream and downstream phases.

Figure 2 presents a general scheme of the bioherbicide production process based on fungal or bacterial metabolites.

## 3. Overview of Current Microbial Bioherbicides Based on Microbial Cultures and Cell-Free Microbial Metabolites

Currently, there are both commercial and non-commercial biological products based on bacteria and fungi for weed control. Bioherbicides derived from cell-free microbial metabolites, such as peptides, phytotoxins, enzymes, and other compounds, have also been described. These bioherbicides are projected to be more efficient alternatives with greater control potential, although extensive research is still required [21,73]. In the following section, we provide an up-to-date overview of microbial bioherbicides using a literature review approach covering the last ten years. This review aims to gather information on the type of microorganism, type of bioherbicide (microbial culture or cell-free metabolite), and their current status as commercial products, including both bacterial and fungal bioherbicides.

### 3.1. Bioherbicides Based on Bacteria

Bacteria that are associated with bioherbicide products are mostly microorganisms that have been isolated from the weeds themselves or their environment. This group of bacteria is known as plant-associated bacteria (PAB), which also involves soil bacteria adjacent to the rhizosphere, rhizobacteria, endophytic bacteria, and phytopathogenic bacteria that co-evolve with crops and weeds [74]. It is also known that among the most recurrent weed pathogenic microorganisms there are those belonging to the genera *Pseudomonas* and *Xanthomonas*, two Gram-negative bacteria of the class Gammaproteobacteria [30]. Many species of *Pseudomonas* present a wide range of plant hosts and are ranked first among the top 10 phytopathogenic bacteria according to a survey by Molecular Plant Pathology [75].

Table 1 shows that the predominant genus of bacteria with bioherbicidal activity is *Pseudomonas*, which accounts for a total of six recorded cases where a bioherbicide was developed based on microbial metabolites. An example is 2-(hydroxymethyl)phenol, a compound isolated from *P. aeruginosa* C1501 in a study by Boyette et al. (2013) [76]. This metabolite achieved a disease severity percentage of nearly 100% at a dose concentration of 2.5 μg/μL when used against green amaranth (*Amaranthus hybridus*), with no notable effects on sorghum seedlings, thus demonstrating its high specificity for weeds. Additionally, no negative effects were found in the soil after 70 days of trials, leading to the conclusion that this compound is not only efficient but also cost-effective and environmentally friendly for field application. This characteristic is significantly important in the development of bioherbicides, as the main obstacles hindering the progress of these biological treatments include unwanted toxicity, high persistence, and potential spread to other environments [15,21].

In a similar study, Lawrance et al. (2019) [28] isolated a rhizobacterium identified as *P. aeruginosa* H6 with high bioherbicidal activity against *Pennisetum purpureum*, *Oryza sativa*, *Pisum sativum*, and *Amaranthus spinosum*. This species produces quinoline derivatives, such as 1,2-dihydroquinoline, which has been patented for use as a herbicide due to its high phytotoxicity against target weeds [77].

Some bacterial species are pathogenic to both weeds and crops simultaneously. However, these are carefully distinguished based on the pathotype variant “pv.” of the microorganism and the mechanism of host infection [78]. For example, *Pseudomonas syringae* pv. tomato affects tomato crops by multiplying in the apoplasts of the leaves and other plant tissues, utilizing the host cells’ nutrients to produce proteins and effectors that increase bacterial colonization and cause damage, leading to bacterial spot of tomato [79]. Conversely, *Pseudomonas syringae* pv. *tagetis* is a pathogen of several weeds of the Asteraceae family [80] and crops such as *Ambrosia tuberosa* and *Helianthus annuus* [81]. Its mechanism of action involves the production of a toxin called tagetitoxin, which inhibits RNA polymerase in chloroplasts, preventing the accumulation of ribulose 1,5-bisphosphate carboxylase and leading to chlorosis in the tissues of developing shoots [82,83].

In recent years, multiple pathogenic variants of both *Pseudomonas* and *Xanthomonas* have been investigated, with promising results at the laboratory and greenhouse levels. *Pseudomonas fluorescens* D7 is a strain with herbicidal activity for controlling downy brome (Bromus tectorum) and other winter annual weeds [84]. Verdesian Life Sciences, LLC, Cary, NC, registered this herbicide as a commercial product in 2014 [85]. Its effectiveness was tested at the laboratory level many years before its commercial launch, showing promising results in reducing annual weed roots in petri dishes and treatment chambers [86]. At the field level, it reduced the seed yield by 16–64%, plant density by 0–35%, and plant shoot mass by 0–54% [87]. However, its efficiency was questioned in a subsequent field study conducted in Wyoming, USA, which compared its efficacy in controlling downy brome to a synthetic herbicide (Imazapic) and found no significant weed reduction compared to the control and the synthetic herbicide [88]. *Pseudomonas fluorescens* BRG100 is another strain with bioherbicide activity that is still under development for commercialization. It is useful for controlling different types of weeds, such as green foxtail (*Setaria viridis*), foxtail barley (*Hordeum jubatum*), crabgrass (*Digitaria sanguinalis*), and annual ryegrass (*Lolium rigidum*). This strain was developed by Agriculture and Agri-Food Canada (AAFC) under U.S. patent 6,881,567, showing 85–90% efficacy for weed control by inhibiting seed germination and suppressing root growth [89]. Laboratory tests are constantly innovated and improved, with experiments conducted using both the crude culture of the microorganism and the cell-free compounds produced by these bacteria.

Regarding bioherbicides based on bacteria of the genus *Xanthomonas*, a review of the last ten years of literature records only two laboratory-scale research studies. Boyette and Hoagland (2013) [90] isolated a bacterial strain identified as *Xanthomonas* spp. LVA987 from diseased leaf tissues collected from common cocklebur (*Xanthium strumarium* L.), an economically important weed that affects soybean plantations in different countries and is resistant to chemical herbicides [91,92]. Trials revealed that *Xanthomonas* spp. LVA987 achieved a mortality rate of 98% against common cocklebur and 80% against horseweed (*Conyza canadensis* L). Subsequently, the same authors studied the effects of environmental parameters on the bioherbicidal activity of *Xanthomonas* spp. LVA987, later identified as *Xanthomonas campestris*, against *Conyza canadensis* L. at the greenhouse level. Plant mortalities of 80% and 60% were achieved at the rosette and bolting growth stages, respectively, when a dew period was applied at 25 °C [93]. Both trials demonstrate the specific infective capacity of *Xanthomonas campestris*, but more recent reports on cell-free metabolites are not yet available.

The strain *X. campestris* pv. poae (JT-P482), first registered in Japan, became the basis of the first commercial bioherbicide product composed of bacteria under the name Camperico^TM^ and was subsequently patented for the specific control of the weeds *Poa annua* and *Poa attenuata* [94,95]. However, there is currently no information on whether this product is commercially active [21]. One of the few reports on bioherbicide activity based on cell-free metabolites is a phytotoxin obtained from *Xanthomonas retroflexus*, composed of a mixture of minor molecular compounds, including organic acids and cyclo-(proline-phenylalanine). These compounds present specific inhibitory activity against dicotyledonous weeds (*Amaranthus retroflexus*, *Capsella bursa*-*pastoris*, and *Portulaca oleracea*), with up to 83.6% root length reduction [66]. Despite these optimistic results, follow-up studies are still lacking.

Another bacterial genus with a considerable variety of microbial metabolites exhibiting bioherbicidal activity is Streptomyces, a group of filamentous bacteria. Metabolites such as Thaxtomin A, diethyl 7-hydroxytrideca-2,5,8,11-tetraenedioate, N-phenylpropanamide, and cinnoline have shown significant effects against common weeds such as *Digitaria sanguinalis* (southern crabgrass) and *Lamium amplexicaule* (henbit). Other bacteria, albeit fewer in number, belonging to the genera *Enterobacter*, *Bacillus*, and *Serratia* have also been recorded. It is noteworthy that only Thaxtomin A has been registered as a microbial metabolite-based bioherbicide available on the biopesticide market, although it can also be synthesized chemically [96].

**Table 1:** Bacterial genera and species with bioherbicide potential based on scientific reports from the last ten years and their status as commercial products.

Microbial Genus	Bacterial Species	Target(s)	Type of Bioherbicide		Status/Commercial Product	Reference
			Microbial Cultivation	Cell-Free Metabolite		
*Bacillus*	*Bacillus flexusmediante* JMM24	*Lathyrus aphaca* L	-	5-aminolevulinic acid	Unavailable	[97]
	*Bacillus* sp. 6	*Anagallis arvensis* L., *Phalaris minor* Retz., *Cynodon dactylon* L., *Melilotus indicus* L.	Microbial culture	-	Unavailable	[98]
	*Bacillus* sp. KA37	*Setaria glauca*	Microbial culture	Cellulase	Unavailable	[99]
	*Bacillus* sp. TR25	*Amaranthus palmeri* S. Wats	Microbial culture	Microbial metabolite (UN)	Unavailable	[32]
*Enterobacter*	*Enterobacter* sp. I-3	*Echinochloa crus*-*galli* L., *Portulaca oleracea* L.	Microbial culture	Indole-3-acteic acid	Unavailable	[100,101]
	*Enterobacter* sp. TR18	*Amaranthus palmeri* S. Wats	Microbial culture	Microbial metabolite (UN)	Unavailable	[32]
*Pseudomonas*	*Pseudomonas aeruginosa* B2	*Amaranthus hybridus* L., *Echinochloa crus*-*galli* (L.) Beauv.	-	Microbial metabolite (UN)	Unavailable	[102]
	*Pseudomonas aeruginosa* C1501	*Amaranthus hybridus*	-	2-(hydroxymethyl) phenol	Unavailable	[76]
	*Pseudomonas aeruginosa* CB-4	*Digitaria sanguinalis*	-	Microbial metabolite (UN)	Unavailable	[103]
	*Pseudomonas aeruginosa* H6	*Pennisetum purpureum*, *Oryza sativa*, *Pisum sativa*, *Amaranthus spinosum*	-	Microbial metabolite (UN)	Unavailable	[28,77]
	*Pseudomonas fluorescens* 6 K	*Anagallis arvensis* L., *Phalaris minor* Retz., *Cynodon dactylon* L., *Melilotus indicus* L.	Microbial culture	-	Unavailable	[98]
	*Pseudomonas fluorescens* ACK55	*Bromus tectorum* L., *Aegilops cylindrica* L., *Taeniatherum* caput-*medusae* L.	Microbial culture	-	Unavailable	[104]
	*Pseudomonas fluorescens* biovar A strain LRS12	*Poa annua* L.	Microbial culture	-	Unavailable	[105]
	*Pseudomonas fluorescens* biovar B strain XJ3	*Poa annua* L.	Microbial culture	-	Unavailable	[105]
	*Pseudomonas fluorescens* biovar B strain XS18	*Poa annua* L.	Microbial culture	-	Unavailable	[105]
	*Pseudomonas fluorescens* BRG100	*Setaria viridis*, *Hordeum jubatum*, *Digitaria sanguinalis*, *Lolium rigidum*	Microbial culture	-	Unavailable	[89,106]
	*Pseudomonas fluorescens* D7	*Bromus tectorum*	Microbial culture	-	Available/D7^®^	[86,87,88,107,108]
	*Pseudomonas fluorescens NKK78*	*Bromus tectorum* L., *Aegilops cylindrica* L., *Taeniatherum* caput-*medusae* L.	Microbial culture	-	Unavailable	[104]
	*Pseudomonas fluorescens SMK69*	*Bromus tectorum* L., *Aegilops cylindrica* L., *Taeniatherum* caput-*medusae* L.	Microbial culture	-	Unavailable	[104]
	*Pseudomonas* sp. TR10	*Amaranthus palmeri* S. Wats	Microbial culture	Microbial metabolite (UN)	Unavailable	[32]
	*Pseudomonas* sp. TR36	*Amaranthus palmeri* S. Wats	Microbial culture	Microbial metabolite (UN)	Unavailable	[32]
	*Pseudomonas trivialis* X33d	*Bromus diandrus*	Microbial culture	-	Unavailable	[109]
*Serratia*	*Serratia marcescens* Ha1	*Digitaria sanguinalis*	Microbial culture	-	Unavailable	[110]
*Streptomyces*	*Streptomyces anulatus* strain-329	*Digitaria sanguinalis*, *Sorghum bicolor*	-	C15H23NO5+Na, denominated as 329-C3	Unavailable	[111]
	*Streptomyces anulatus* UTMC 2102	*Cardaria draba*	-	Microbial metabolite (UN)	Unavailable	[112]
	*Streptomyces olivochromogenes* KRA17-580	*Digitaria ciliaris*	-	Cinnoline-4-carboxamide; cinnoline-4-carboxylic acid	Unavailable	[113]
	*Streptomyces scabies*	*Lamium amplexicaule*, *Taraxacum officinale*, *Sherardia arvensis*, *Poa annua* L., *Lolium perenne* L., *Digitaria ischaemum*	-	Thaxtomin A	Available/Opportune^TM^	[114,115,116,117]
	*Streptomyces* sp. DDBH019	*Echinochola crusigalli* L., *Amaranthus spinosus* L., *Cyperus rotundus* L.	-	Diethyl 7-hydroxytrideca-2, 5, 8, 11-tetraenedioate	Unavailable	[118]
	*Streptomyces* sp. KA1-3	*Cassia occidentalis* L., *Cyperus rotundus* L.	-	N-fenilpropanamida	Unavailable	[119]
	*Streptomyces vinaceusdrappus* UTMC 2104	*Cardaria draba*	-	Microbial metabolite (UN)	Unavailable	[112]
*Xanthomonas*	*Xanthomonas campestris* LVA987	*Conyza canadensis*	Microbial culture	-	Unavailable	[93]
	*Xanthomonas campestris* pv. *poae* JT-P482	*Poa annua*, *Poa attenuate*	Microbial culture	-	Available/CampericoTM	[94,95]
	*Xanthomonas retroflexus* L4	*Amaranthus retroflexus*, *Capsella bursa-pastoris*, *Portulaca oleracea*	-	Microbial metabolite	Unavailable	[66]
	*Xanthomonas* spp. LVA987	*Xanthium strumarium*, *Conyza canadensis*	Microbial culture	-	Unavailable	[90]

UN: Uncharacterized.

### 3.2. Bioherbicides Based on Fungus

Fungi represent a significant biological group that can be utilized as a biotechnological tool for weed control. Notably, there are currently more studies on potential or commercial bioherbicide products derived from fungi than those based on bacteria [35]. Fungi associated with plants can exert both beneficial and detrimental effects. Among the approximately 150,000 described fungal species, around 8000 are phytopathogenic [120]. Similar to plant-associated bacteria (PAB), these phytopathogenic fungi can be harnessed to direct their inhibitory activity against weeds that affect crops. One primary mechanism by which fungi initiate plant infection is through the production of enzymes that degrade the plant cell wall. These enzymes hydrolyze polysaccharides in the cell wall, facilitating fungal colonization and nutrient acquisition essential for metabolic growth [121]. Additionally, fungi employ specific mechanisms, such as the production of mycotoxins or hormones, which confer a more selective antagonistic profile, a crucial factor in herbicide development [31].

A comprehensive review of bibliographic studies from the past decade reveals a notable increase in the diversity of fungi with bioherbicidal potential, particularly in terms of the genus and species. The genus *Alternaria* and its various species, such as *Alternaria cassia* and *Alternaria sonchi*, are prominent. *Alternaria sonchi* has been reported to produce a microbial metabolite with bioherbicidal properties against *Sonchus arvensis*, commonly known as field milk thistle [122]. Additionally, the genus *Phoma*, including species such as *Phoma dimorpha* and *Phoma macrostoma*, shows considerable bioherbicidal potential [123]. Furthermore, the genus *Trichoderma* has been extensively documented as a bioherbicidal alternative. Studies highlight both microbial cultures and cell-free metabolites, such as the ethyl ester of linoleic acid, which can inhibit the growth of the weed *Echinochloa colona* [124]. Moreover, amylase and cellulase enzymes from *Trichoderma* have proven effective in *Cucumis sativus* [40].

According to Duke (2023) and Roberts et al. (2022) [21,23], only a few commercial fungus-based bioherbicide products are available on the market. Notable examples include LockDown^®^, a bioherbicide based on *Colletotrichum gloeosporioides* f. sp. *aeschynomene*, developed for controlling northern snow pea (*Aeschynomene virginica*). Initially registered in 1982 as Collego^TM^, it was reintroduced in 2006 under EPA registration number 82681-1 [125,126]. Bio-Phoma™ is a bioherbicide made from *Phoma macrostoma*, effective against many broadleaf weed species, and is registered with the Pest Management Regulatory Agency of Canada (PMRA) for domestic and commercial use [127,128]. Di-Bak^TM^ Parkinsonia, based on the fungi *Lasiodiplodia pseudotheobromae* NT039, *Macrophomina phaseolina* NT094, and *Neoscytalidium novaehollandiae* QLD003, is commercially active for controlling *Parkinsonia aculeata* and is the only product available for woody weeds [129]. Kichawi Kill™, derived from *Fusarium oxysporum* f. sp. *strigae*, targets witchweed (*Striga hermonthica*), a pest causing significant yield losses in maize across over 200,000 hectares in Kenya alone [130]. This product is currently under study for performance improvement through genetic engineering and optimization of the application method, thus its commercial production is still limited [131].

In the research domain, advancements in technology and the development of new techniques have led to yield improvements and progress in various aspects of using biological herbicides for weed control. For fungi of the *Alternaria* genus, it is known that they produce over 300 metabolites, some of which exhibit bioherbicidal activity, with a few displaying host specificity—a key requirement for effective weed control agents [132]. Dalinova et al. (2020) [133] presented a list of *Alternaria* species with bioherbicidal potential, highlighting *Alternaria alternata* as a specific pathogen for the weeds *Echinochloa* spp. and *Eichhornia crassipes* [134,135]. *Alternaria macrospora* targets *Parthenium hysterophorus* [136], and *Alternaria sonchi* targets *Sonchus arvensis* [122], both of which are economically significant due to their high efficiency.

The genus *Trichoderma* is emerging as a promising candidate for biological weed control. Zheng et al. (2023) successfully isolated six *Trichoderma* species, including *Trichoderma koningiopsis*, *Trichoderma afroharzianum*, *Trichoderma atroviride*, *Trichoderma virens*, and *Trichoderma asperelloides*. The study demonstrated that the ethyl ester of linoleic acid exhibited the highest inhibitory activity against the germination and growth of *Echinochloa colona* seeds by altering the regulation of abscisic acid produced by the plant through volatile organic compounds (VOCs) [124]. Additionally, crude fungal cultures and cell-free filtrates of *T. koningiopsis* exhibit high bioherbicidal activity against weeds of the genus *Euphorbia heterophylla*, *Bidens pilosa*, and *Conyza bonariensis* [137,138,139]. For *E. heterophylla*, Bodin et al. (2018) achieved 60–90% phytotoxicity of the leaf area using the crude biocompound, compared to 0–50% with filtered compounds, after optimizing the culture medium [140]. A more recent study by Ulrich et al. (2023) evaluated the bioherbicidal effect of the cell-free supernatant of *T. koningiopsis* in combination with varying concentrations of chemical herbicides, achieving 100% control of *E. heterophylla* with only half the recommended concentration of the commercial herbicide. This combined application reduces the likelihood of resistance development, particularly in species like *E. heterophylla*, where resistance has already been reported in Brazil [141].

The genus *Fusarium* is another significant group of fungi for biological weed control. There is scientific evidence of their phytopathogenic traits, allowing them to act as pathogens affecting crops or as bioherbicides. An early report by Boyette et al. (1993) demonstrated the potential of *Fusarium* biomass as a bioherbicide, controlling 95%, 98%, and 80% of *Cassia occidentalis*, *Senna obtusifolia*, and *Sesbania herbacea*, respectively [142]. Later, Nzioki et al. (2016) tested the phytotoxicity of *Fusarium oxysporum* against *Striga hermonthica*, identifying tyrosine as the amino acid most effective in reducing the biomass of *Striga hermonthica* to 1 g. Subsequently, a product based on *Fusarium oxysporum* f. sp. *strigae* (Kichawi Kill™) was formulated and approved for use by the Kenya Pest Control Products Board in 2021 [131]. To enhance the product efficiency, the authors developed an application method involving seed coating, resulting in 88–93% phytotoxicity in field trials.

Other fungal groups also demonstrate significant efficiency, making them highly relevant for further study to explore additional commercial production alternatives. Below is an up-to-date table featuring the most relevant studies on fungal bioherbicides or metabolites produced by fungi over the last ten years (Table 2).

## 4. Limiting Factors and Innovative Solution Alternatives

According to the current status of bioherbicides derived from microorganisms such as bacteria and fungi, the prevailing perception is that of limited or almost negligible commercial production. This differs greatly from the significant research landscape, where numerous proposals for biological weed control have been explored. Despite the multitude of studies conducted, there is currently limited interest and diminished investment potential in such endeavors within the market. As previously mentioned, and in agreement with Duke (2023) and Roberts et al. (2022) [21,23], the products currently marketed are not sufficient in volume to deal with weed control, especially given the current issue of resistance to chemical herbicides, which is increasing day by day [9].

To understand some of the key reasons for the challenges facing bioherbicides today, it is important to consider one of the most significant factors: the range of weed species that a bioherbicide can effectively target compared to a chemical herbicide. Generally, a chemical herbicide has a wide range of actions, meaning it is efficient against more than one type of weed and is economically more attractive to buyers, as multiple weed types can coexist in a crop field. In the case of biological herbicides, they have a narrow and often selective range, which can be problematic for large-scale treatments. Certain groups of biological controllers exhibit a broad target range, particularly for weeds closely related taxonomically. However, this wide spectrum of action is not commercially viable, as these products could persist in the environment and potentially impact non-target crops. Thus, the viability of bioherbicides at the commercial level will be strictly linked to the formulation of products with neither too broad nor too limited a spectrum [52].

Persistence, as already mentioned, is another limiting factor associated with bioherbicides, especially those composed of living cells. Weed phytopathogens, upon encountering their target, will utilize all the available nutritional resources for their development. Once these resources are depleted, the microorganisms will seek additional resources within the ecosystem, including those from surrounding crops or at the rhizosphere level. They may even form survival structures such as spores, which have a greater ability to withstand stressful conditions, increasing the likelihood of spreading to neighboring crops, which may become new targets for phytopathogens.

Storage and application are also factors influencing bioherbicides’ efficiency as biological weed controllers and can make them less attractive for field application. Unlike chemical inputs, microorganisms must remain viable, meaning they must stay alive or replicate in an environment to guarantee their efficiency. Multiple factors affect microorganisms’ viability, including physicochemical conditions such as the temperature and pH, and biological conditions such as random mutations due to metabolic stress, which can inhibit or suppress their ability to produce a phytopathogenic metabolite of interest [167,168]. Therefore, the formulation of herbicides must be strictly controlled to ensure their efficiency; otherwise, they will have no effect. Similarly, application plays an important role, considering various environmental factors not accounted for in laboratory tests.

Negative aspects have been reported in studies such as that by Tekiela (2018) [85], who used D7, a commercial bioherbicide product based on *Pseudomonas fluorescens* D7, for controlling *Bromus tectorum* at the field level, with no positive results compared to synthetic commercial herbicides. Pike et al. (2020) [107] also reported the inefficiency of D7 when tested through direct spray application on *B. tectorum* over 162 hectares. Beyond these studies, bacterial strains such as *P. fluorescens* ACK55 against *Bromus arvensis* L. and *P. fluorescens* MB906 against *Taeniatherum caput-medusae* [108,169], as well as fungi like *Alternaria destruens* for controlling *Cuscuta* spp. [144], *Colletotrichum acutatum* for controlling *Hakea sericea* [170], and *Colletotrichum gloeosporioides* f. sp. *malvae* for controlling *Malva pusilla* [171], have been discontinued due to their low viability for weed control.

Given the factors diminishing the viability of bioherbicides and their limited availability or inconsistent distribution in the commercial sector, various alternative solutions to address the current issues are under study and research.

**Figure 3:**
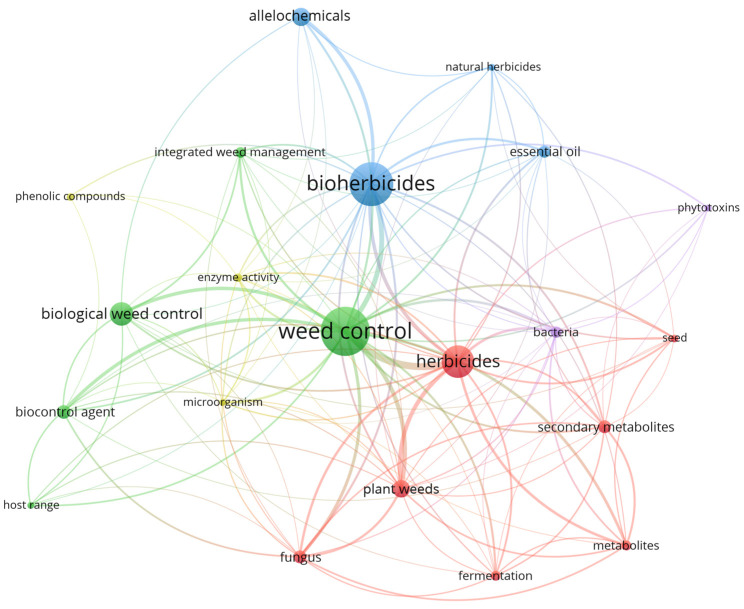
Network analysis of scientific terms used to identify innovative trends in the field of microbial bioherbicides. The scientific landscape was developed using VOS Viewer.

To examine the current landscape of microbial bioherbicides in bibliographic terms, a systematic search was conducted in the SCOPUS bibliographic database. The following search algorithm was employed: TITLE-ABS-KEY (“microbial herbicides” OR “microbial bioherbicides” OR “bioherbicides” OR “Microbial herbicidal agents” OR “Biological weed control” OR “Biological herbicides” OR “Microbial weed control” OR “Biological weed management agents” OR “Herbicidal microorganisms” OR “Microbial weed organisms”) AND PUBYEAR > 2013 AND PUBYEAR < 2025. Consequently, a total of 496 documents related to this field of study over the past 10 years were identified. These documents were filtered based on the most relevant scientific terms to conduct a network analysis and determine the main innovation trends regarding bioherbicides (Figure 3).

The analysis reveals that the larger nodes, such as “Bioherbicides”, “Weed Control”, and “Herbicides”, exhibit an integral correlation with the smaller nodes. Each related term is represented by a distinct color, grouped in clusters, and connected by thicker lines. This indicates a high degree of coincidence between two or more terms in a scientific article. Similarly, it can be observed that metabolites or secondary metabolites appear as a trend in reports associated with fungal and bacterial treatments. In other clusters, additional components emerge, including phytotoxins associated with bacteria, microorganisms and their enzymatic activity, phenolic compounds, allelochemicals, and technologies such as integrated weed management, biological control of weeds, and other biocontrol agents.

Based on this analysis, one of the main proposals to improve the current panorama of bioherbicides is to focus on their use as a cell-free preparation using only the metabolites produced by the candidate phytopathogens. This approach addresses a key drawback, namely the restrictions imposed by regulatory agencies in each country on the use of microorganisms that may spread to non-target plants, particularly crops near the application area. For example, in Brazil, the regulatory agencies for the use of bioherbicides as agrochemical products are ANVISA (Agência Nacional de Vigilância Sanitária), IBAMA (Instituto Brasileiro do Meio Ambiente e dos Recursos Naturais Renováveis), and MAPA (Ministério da Agricultura, Pecuária e Abastecimento). These agencies are responsible for regulating aspects related to public health and toxicological evaluation of the product, environmental regulation and possible environmental impacts, and the registration of these products, verifying that they comply with regulations and agronomic efficacy, respectively.

In the same context, regulations in Europe are increasingly in favor of the use of biological pesticides. The European Commission’s Pesticides Headline Goal Framework aims to reduce pesticide use by half by 2030. This strategy includes actions such as redesigning farming systems, improving prophylaxis, and supporting the development of public policies and private initiatives for the transition to pesticide-free agri-food systems. Additionally, reducing certain requirements for biopesticide registration, such as toxicological testing, environmental testing, residual testing, and acute oral and dermal testing, could facilitate their development [172].

Although biopesticides are generally permitted for use, their widespread adoption is hindered by the challenge of controlling the persistence of live cell-based microbial bioherbicides in the environment. In this regard, bioherbicides based on cell-free microbial metabolites may offer a significant advantage.

Likewise, the use of phytopathogenic microbial metabolites solves to a great extent the problem of product persistence in the environment, since it is not a living organism and the metabolites can be easily biodegraded by other species in the microbiota or by abiotic factors in the ecosystem. This allows for crop rotation, a common practice for the improvement of soil health, without the risk of the existence of a phytopathogen that indirectly affects the new crops. For example, Chaves Neto et al. (2021) [25] used metabolites produced by *Phoma dimorpha*, obtained by ultrafiltration, microfiltration, and nanofiltration, for the control of *Echinochloa* sp., *Amaranthus cruentus*, *Senna obtusifolia*, and *Bidens pilosa*. Phytotoxicity levels of up to 100% were reached, demonstrating not only the high phytotoxic efficiency of the metabolites produced by *Phoma dimorpha* but also the high potential of the membrane separation process, which can be considered one of the most efficient techniques in the downstream process. Additionally, metabolites can also be produced in volatile forms, as demonstrated by Zheng et al. (2023) [124] in their studies with *Trichoderma* spp. isolated from rhizospheric soil. This species produces phenylethyl alcohol, 2,4,6-trichloroanisole, hexadecanoic acid ethyl ester, 10(E),12(Z)-conjugated linoleic acid, and linoleic acid ethyl ester, which inhibited the growth of *Echinochloa colona*, directly affecting the hormonal regulation of the weedy plant. Several scientific findings [58,173,174,175,176,177,178] offer promising results [173,174,175,179,180,181,182]. However, further studies are necessary to verify whether the production of metabolites is exclusively induced by their host. In such a scenario, fermentation processes would need to incorporate the target weeds as components of their culture media to effectively stimulate the gene regulation associated with the production of the desired metabolites.

Similarly, in order to enhance the use of cell-free metabolites as bioherbicides, improvements must be made to the product formulation and application. As evidenced by the findings of this review, these two factors also impede the development of biological herbicides in the market. Multiple environmental factors can reduce the efficiency of a biological herbicide, particularly when working with products containing live microorganisms. For example, in terms of the formulation, Namasivayam et al. (2023) [35] tested biogranular preparations and microemulsions based on natural products, improving the herbicidal activity against *Amaranthus retroflexus*. In addition to efficiently adhering spores of *Alternaria alternata*, *Paecilomyces* sp., *Fusarium oxysporum*, and *Aspergillus niger*, this strategy also admitted the phytotoxic metabolites produced by these, generating antibacterial activity against *Pseudomonas syringae* pv. *syringae*, a phytopathogen of several commercially important crops. Studies have demonstrated that incorporating both commercial surfactants, like Tween 80, and biological surfactants, such as rhamnolipid, enhances the permeability of bioherbicide active agents, facilitating their entry into target plant cells [72,160]. This presents an even more economically favorable scenario, as it is well documented that the same microbial strains exhibiting herbicidal activity also possess the capability to produce biological surfactants [176]. Consequently, this would result in a more viable alternative in product formulation, saving steps in the search for additional adjuvants.

On the other hand, the application of bioherbicides as a coating on crop seeds has also demonstrated high efficiency, especially in a preventive way, limiting weed growth. This method has even been observed to enhance the crop yields during growth, without negatively altering surrounding crops or the ecosystem [131,177]. Similarly, utilizing encapsulation techniques with micro- or nanotechnology can address issues like the stability of bioherbicide compounds in the environment. While bioherbicides are eco-friendly and reduce environmental concerns, ensuring they remain effective over time can be challenging. Encapsulation helps by allowing controlled release, improving the effectiveness while maintaining environmental safety [167,178].

The analysis also suggests that integrated management for the control of weeds may be a crucial step toward improving efficiency. In a study published in 2023, Ulrich et al. [38] demonstrated that employing bioherbicides alongside chemical herbicides at minimal concentrations can enhance the efficiency by up to 100% in terms of weed control. This approach offers multifaceted benefits, as it demonstrates a positive synergistic effect while also reducing the risk of resistance mechanisms in weeds. These interactions have been proven in metabolites produced by fungi of the genus *Trichoderma* sp., *Colletotrichum*, *Phoma*, and *Alternaria*, in conjunction with the chemical herbicides glyphosate, glufosinate-ammonium, 2,4-dichlorophenoxyacetic acid, thidiazuron, and 4-D plus MCPP (2-[4-chloro-2-methylphenoxy]propanoic acid), for the control of the weeds *Euphorbia heterophylla*, *Brachiaria plantaginea*, *Bidens pilosa*, *Conyza bonariensis*, *Abutilon theophrasti*, *Convolvulus arvensis*, and *Senna obtusifolia* [38,183].

### Patent Analysis on Microbial Bioherbicides

To assess the historical progression of patents in microbial bioherbicide production, the Derwent Innovations Index^®^ database and INPI (Instituto Nacional da Propriedade Industrial, Brazil) were consulted. The keywords used in the search were: TS = ((bioherbicide or bio-herbicide or bio-herbicides or biological* herbicides*) not (extracts) not (essential* oil*) not (nicotine*) not (terpenes*) not (botanical*) not (chemical herbicides) not (Agrochemical) not (solvent) not (aromatic) not (transgenic plant) not (fertilizer) not (glyphosate)) and (bioherbicidas or bio-herbicides) for Derwent Innovations and INPI, respectively.

**Figure 4:**
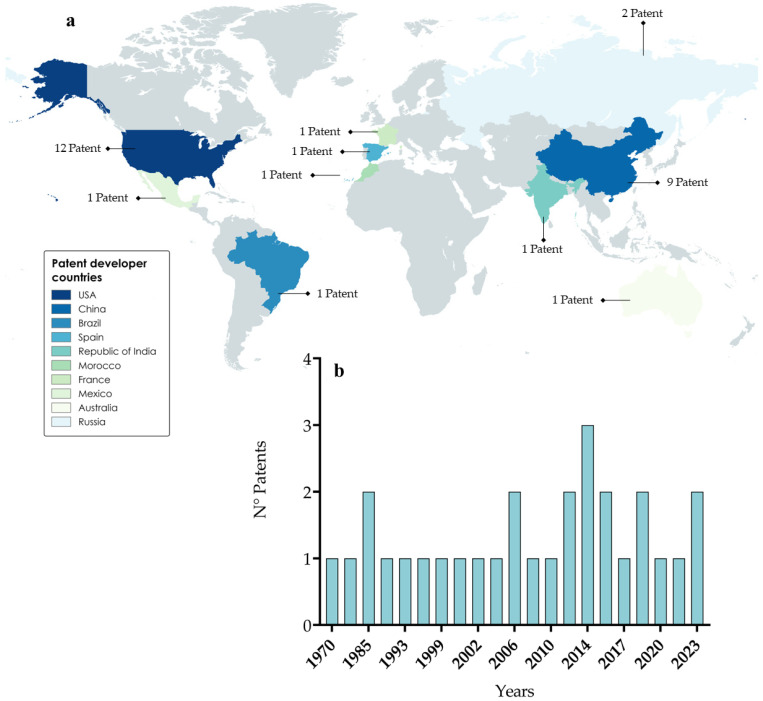
(**a**) World distribution of patents related to herbicides from microorganisms. (**b**) Patent distribution over the years.

The documents were exported to MS Excel^®^ and subsequently manually selected by analyzing the title and summary of each work. In total, 30 patent documents were selected. The primary holders of patents for bioherbicide production technology with microorganisms are the United States and China, accounting for 40% and 30% of patents, respectively. A plausible explanation for this is that these countries have historically been agricultural potencies, leading to advancements in this field being safeguarded through patents [184]. Other countries are listed in Figure 4.

Furthermore, an increasing trend was observed in the number of patents filed per decade. For example, in the 2000s, 2010s and 2020s (still 6 years to go) 26%, 33% and 16% of patent filings were made, respectively. This can be explained by the growing interest in natural or organic products due to their lower or absent impact on the environment, as well as on human and animal health [185].

The first patent application for bioherbicides with microorganisms dates back to 1972 in the Soviet Union (SU343671A1). This patent presents a process for the preparation of a bioherbicide and a process for the control of the weed *Ambrosia artemisiifolia* using *Albugo tragopogonis*. *A. artemisiifolia* plants are treated early (up to the stage of two pairs of leaves) with a suspension of *A. tragopogonis* conidia at a concentration of 10,000 spores/mL. The suspension is applied at a rate of 500 L/ha. According to Zhergin et al. (1972) [186], this method is more effective than any synthetic herbicide. The fungus *A. tragopogonis* develops severe infections in the *A. artemisiifolia*. According to Hartmann and Watson (1980) [187], when *A. artemisiifolia* is treated with *A. tragopogonis* spores, pollen and seed production decreases by 99% and 98%, respectively. Furthermore, it reduces the weight of the plant by around 80%.

Walker (1983) [188], in invention US4390360A, presented a process for the biological control of sicklepod, showy crotalaria, and coffee senna diseases using the fungus *Alternaria cassiae*, which can produce typical lesions on the mentioned herbs. The process involves inoculating the fungus into the herbs, applying the biological agent via spraying or wettable powder using an inert vehicle, which can be vermiculite, clay, or corn cob grains. The production of conidia was carried out by submerged fermentation and the growth medium was composed of juice (200 mL/L), calcium carbonate (3 g/L) and sucrose (30 g/L). The process was carried out at 26 °C and the mycelia were harvested between 48 and 72 h after inoculation.

After harvesting, the mycelia were exposed to direct light for 20 to 30 min in chambers to stimulate sporulation. Subsequently, the mycelia were transferred to unilluminated cameras. Finally, the spores were collected and mixed with vermiculite until reaching a concentration of 10^5^ conidia/g vermiculite.

Jin and Custis (2011) [189], in patent WO2011156109A1, presented a process for encapsulating fungal spores to be used for agricultural purposes. According to the authors of this invention, the encapsulation process improves the survival rate of conidia by more than 90%, increasing the efficiency of the bioherbicide. For the encapsulation process, a sucrose solution must be used in a concentration of 0.1 to 10 (% *w*/*v*), in which the conidia are suspended. The role of sucrose is to protect the microbial spores as a cryoprotectant. The temperature is the main variable in this process and thus requires careful control. In the spray dryer and in the output stream, the temperature must be maintained in the ranges 40 °C to 140 °C and 20 °C and 80 °C, respectively. This process can be used to prepare spores of the following microorganisms: species of *Bacillus*, *Entomoph*, *Paecilomyces*, *Metarhizium*, *Beauveria*, *Penicillium*, *Trichoderma*, *Steinernema*, *Heterorhabditis* or mixtures thereof.

Chen et al. (2023) [190] in their patent disclosed a new strain, *Talaromyces purpureogenus* CY-1, which can produce metabolites with herbicidal activity. These metabolites are capable of controlling broadleaf weeds such as *Amaranthus retroflexus*, *Alternanthera sessilis*, *Chenopodium album*, and *Conyza canadian*, among others. The microorganism grows in PD (Potato Dextrose) medium at a temperature between 25 °C and 28 °C, and the fermented broth is separated from the cellular biomass by filtration. Once the broth is sprayed on the herbs, they start to show symptoms of phytotoxicity. However, this bioherbicide does not have a significant herbicidal effect against grasses. According to the inventors, the metabolites produced by *T. purpureogenus* can damage more than 85% of the plant’s structure in 7 days, which causes the plant’s death. Other patents are summarized in Table 3.

## 5. Conclusions

Microbial bioherbicides offer promising potential as an alternative for weed control. However, recent reports on the current state of their commercial production and patents are scarce compared to other biologically based products in various industrial sectors. Conversely, the scientific literature presents numerous novel herbicidal alternatives developed in the past decade, prominently featuring bioherbicides formulated from cell-free microbial metabolites. This disparity reflects industry reluctance to invest in biocontrol agents, historically justified by their lower efficacy compared to chemical products, high production costs, and lack of specificity. Nevertheless, as discussed in this review, the emerging trend toward microbial bioherbicides using cell-free phytotoxic metabolites could offer a potential solution to these challenges. Notably, microbial genera such as *Pseudomonas*, *Xanthomonas*, *Bacillus*, and *Streptomyces* for bacteria, and *Alternaria*, *Fusarium*, *Phoma*, and *Trichoderma* for fungi, are highlighted as significant candidates for studying the production of these phytotoxic metabolites of interest. Moreover, the use of agro-industrial residues as fermentation substrates could alleviate production costs, transforming these products into economically viable options for companies. Advances encompass all the stages, from strain development to final product formulation and application techniques, which could facilitate wider distribution and application of bioherbicides, thereby achieving more effective and affordable weed control. This addresses a significant agricultural challenge and mitigates considerable economic losses.

## Figures and Tables

**Table 2:** Fungal genera and species with bioherbicide potential based on scientific reports from the last ten years and their status as commercial products.

Microbial Genus	Fungal Species	Target(s)	Type of Bioherbicide		Commercial Product/Status	Reference
			Microbial Cultivation	Cell-Free Metabolite		
Albifimbria	*Albifimbria verrucaria*	*Conyza canadensis*	Microbial culture	-	Unavailable	[143]
Alternaria	*Alternaria alternata*	*Amaranthus retroflexus*	Microbial culture	-	Unavailable	[35]
	*Alternaria alternata* AL-14	*Eichhornia crassipes*	Microbial culture	-	Unavailable	[134,135]
	*Alternaria cassia*	*Senna obtusifolia*	Microbial culture	-	Unavailable	[27]
	*Alternaria destruens*	Different species of the *Cuscuta* genus	Microbial culture	-	Unavailable	[144]
	*Alternaria sonchi*	*Sonchus arvensis*	-	Methyl 8-hydroxy-3-methyl-4-chloro-9-oxo-9H-xanthene-1-carboxylate	Unavailable	[122]
Aspergillus	*Aspergillus niger*	*Amaranthus retroflexus*	Microbial culture	-	Unavailable	[35]
	*Aspergillus* sp.	*Ageratina adenophora*	-	Citrin	Unavailable	[145]
Bipolaris	*Bipolaris yamadae* HXDC-1–2	*Echinochloa crus-galli*, *Setaria viridis*, *Leptochloa chinensis*, *Eleusine indica*, *Pseudosorghum zollingeri*, *Leptochloa panicea*, *Bromus catharticus*	Microbial culture	-	Unavailable	[146]
Chondrostereum	*Chondrostereum purpureum*	Hardwoods and deciduous trees and shrubs	Microbial culture	-	Available/Chontrol™; EcoClear™; MycoTech™	[147,148]
Cochliobolus	*Cochliobolus australiensis* (LJ3B1, 2MG2F, LJ3B2, LJ3C1, SNM4C1 y LJ4B)	*Cenchrus ciliaris*	-	Radicinin/dihydropyranopyran-4,5-dione	Unavailable	[149]
Colletotrichum	*Colletotrichum acutatum*	*Hakea sericea*	Microbial culture	-	Unavailable/Hakatak^®^	[150]
	*Colletotrichum gloeosporiodides* f. sp. *aeschynomene*	*Aeschynomene virginica*	Microbial culture	-	Available/LockDown^®^	[125,126,151]
	*Colletotrichum siamense*	*Tridax procumbens*	Microbial culture	-	Unavailable	[152]
Diaporthe	*Diaporthe schini*	*Amaranthus viridis*, *Bidens pilosa*, *E. crus-galli*, *Lollium multiflorum*	Microbial culture	-	Unavailable	[153]
Fusarium	*Fusarium denticulatum*	*C. sativus*	-	Amylase, cellulase, and peroxidase	Unavailable	[40]
	*Fusarium oxysporum*	*Amaranthus retroflexus*	Microbial culture	-	Unavailable	[35]
	*Fusarium oxysporum*	*Avena fatui*	-	Isovitexina, calicosina, quercecetagetina, dihidroxidimetoxiisoflavona	Unavailable	[154]
	*Fusarium oxysporum*	*Ninidam theenjan*	-	Vaeleric acid; 3-(hydroxymethyl)-2-Cyclohexen-1)	Unavailable	[35]
	*Fusarium oxysporum* f. sp. *strigae*	*Striga hermonthica*	Microbial culture	-	Available/Kichawi Kill™	[130,131,155]
	*Fusarium* sp.	*C. sativus*	-	Amylase, cellulase, and peroxidase	Unavailable	[40]
Lasiodiplodia	*Lasiodiplodia pseudotheobromae* NT039	*Parkinsonia aculeata*	Microbial culture	-	Available/Di-Bak^TM^ Parkinsonia	[129]
Macrophomina	*Macrophomina phaseolina* NT094	*Parkinsonia aculeata*	Microbial culture	-	Available/Di-Bak^TM^ Parkinsonia	[129]
Mucor	*Mucor circinelloides*	*C. sativus*	-	Amylase, cellulase, and peroxidase	Unavailable	[40]
Mycoleptodiscus	*Mycoleptodiscus indicus* UFSM 54	*Cucumis sativus*, *Conyza* sp., *Sorghum bicolor*	-	Microbial metabolite (UN)	Unavailable	[24]
Myrothecium	*Myrothecium verucarria*	*Ipomea* spp., *Euphorbia esula*, *Brunnichia ovata*, *Campsis radicans*, *Pueraria lobata*	-	Roridin A and verrucarin A	Unavailable	[156]
Neoscytalidium	*Neoscytalidium novaehollandiae* QLD003	*Parkinsonia aculeata*	Microbial culture	-	Available/Di-Bak^TM^ Parkinsonia	[129]
Nigrospora	*Nigrospora oryzae* YMM4	*Rumex dentatus*, *Sonchus oleraceus*	Microbial culture/microbial metabolite (UN)	-	Unavailable	[157]
Paecilomyces	*Paecilomyces* sp.	*Amaranthus retroflexus*	Microbial culture	-	Unavailable	[35]
Penicillium	*Penicillium sclerotiorum* HY5	*Amaranthus retroflexus* L., *Abutilon theophrasti* M.	-	Sclerotiorins B, ochlephilone, isochromophilone I	Unavailable	[158]
	*Penicillium* sp.	*Ageratina adenophora*	-	Citrin	Unavailable	[145]
Phoma	*Phoma dimorpha*	*Echinochloa* sp., *Amaranthus cruentus*, *Senna obtusifolia*, *Bidens Pilosa*	-	Microbial metabolite (UN)	Unavailable	[25]
	*Phoma macrostoma*	Many species of broadleaf weeds	Microbial culture	-	Available/Bio-Phoma™	[127,128]
	*Phoma multirostrata* TBRC 12769	*Tridax procumbens*	Microbial culture	Norharman and Harman	Unavailable	[159]
	*Phoma* sp.	*Crocus sativus*, *Sorghum bicolor*	Microbial culture	-	Unavailable	[160]
Puccinia	*Puccinia thlaspeos*	*Isatis tinctoria*	Microbial culture	-	Available/Woad Warrior^®^	[161]
Pyricularia	*Pyricularia grisea*	*Cenchrus ciliaris*	-	(10S,11S)-(-)-epi-pyriculol	Unavailable	[162,163]
Sclerotinia	*Sclerotinia minor*	*Araxacum officeinale*	Microbial culture	-	Available/Sarritor™	[164,165]
Trichoderma	*Trichoderma afroharzianum*	*Echinochloa colona*	-	Ethyl ester of linoleic acid	Unavailable	[124]
	*Trichoderma asperelloides*	*Echinochloa colona*	-	Ethyl ester of linoleic acid	Unavailable	[124]
	*Trichoderma atroviride*	*Echinochloa colona*	-	Ethyl ester of linoleic acid	Unavailable	[124]
	*Trichoderma koningiopsis*	*Echinochloa colona*	-	Ethyl ester of linoleic acid	Unavailable	[124]
	*Trichoderma koningiopsis*	*Euphorbia heterophylla*, *Bidens Pilosa*, *Conyza bonariensis*	Microbial culture	Microbial metabolite (UN)	Unavailable	[38,137,140]
	*Trichoderma koningiopsis* MK860714	*C. sativus*	-	Amylase, cellulase, and peroxidase	Unavailable	[40]
	*Trichoderma polysporum* HZ-31	*Elsholtzia densa*, *Polygonum lapathifolium*, *Lepyrodiclis holosteoide*, *Avena fatua*, *Chenopodium album*, *Polygonum aviculare*	Microbial culture	-	Unavailable	[166]
	*Trichoderma virens*	*Echinochloa colona*	-	Ethyl ester of linoleic acid	Unavailable	[124]

UN: Uncharacterized.

**Table 3:** Patented technologies for producing biological herbicides based on bacteria and fungi.

Patent Code	Patent Type	Strain	Technology	Bioherbicides Type	Target Herbs and Effect	Reference
CN116376710-A	Process	*Fusarium proliferatum* APF-1	*Fusarium proliferatum* strain isolation process, application, and separation process	Microbial culture: microbial spore suspension (10^5^ spores/mL)	*Eclipta alba*, after five days of treatment with spores, the plants have yellow, fallen, and rotting leaves.	[191]
CN111436461A	Process	Tea smut disease pathogenic bacteria	Biological herbicide with bacterial powder and nutrient solution	Microbial culture: microbial spore suspension (1.2 × 10^9^ spores/mL)	Horseweed herb, horehound, goosegrass, black nightshade, sticktight, bunge corydalis herb, speedwell, *gynura divaricata*, etc.	[192]
IN201921007625-A	Process	*Phoma* sp. MH595482	Biological synthesis of bioherbicide	Microbial metabolite	*Parthenium hysterophorus*. The metabolites produced by *Phoma* sp. inhibit 100% of herb germination.	[193]
WO2014107107A2	Process	*Streptomyces* sp. N02	*Streptomyces* sp. N02 strain with specified 16S rDNA sequence as a bioherbicide to inhibit seed germination and plant growth	Microbial metabolite: herbicidin and herbimycin	Clover. The compounds inhibit plant growth and produce black spots on plant leaves.	[194]
US5538890A	Process	*Sclerotinia sclerotiorum* (mutant strain)	Broad spectrum biological herbicide	Microbial culture	*Centaurea maculosa* and *Cirsium arvense*	[195]
US4643756A	Process	*Colletotrichum truncatum*	Bioherbicide for Florida beggarweed	Microbial culture: microbial spore suspension (2 × 10^6^ spores/mL)	*Senna obtusifolia*, *Crotalaria spectabilis*, *Senna occidentalis*. The fungus produces critical lesions on seedlings of the trees up to the 3 and 4 leaf stage. The effects on weeds are twisting of the stems, discoloration of the midrib and leaf veins.	[196]

## Data Availability

Data are contained within the article.
